# Research Progress of Protein-Based Bioactive Substance Nanoparticles

**DOI:** 10.3390/foods12162999

**Published:** 2023-08-09

**Authors:** Mengqing Han, Kunlun Liu, Xin Liu, Muhammad Tayyab Rashid, Huiyan Zhang, Meiyue Wang

**Affiliations:** 1 College of Food Science and Engineering, Henan University of Technology, Zhengzhou 450001, China; 2021930498@stu.haut.edu.cn (M.H.); 2021920060@stu.haut.edu.cn (X.L.); trashid208@gmail.com (M.T.R.); 2021920105@stu.haut.edu.cn (M.W.); 2 School of Food and Reserves Storage, Henan University of Technology, Zhengzhou 450001, China; 3 Zhengzhou Ruipu Biological Engineering Co., Ltd., Zhengzhou 450001, China; zhanghuiyan@ruipu.com

**Keywords:** bioactive substances, proteins, nanoparticles, encapsulation, preparation method

## Abstract

Bioactive substances exhibit various physiological activities—such as antimicrobial, antioxidant, and anticancer activities—and have great potential for application in food, pharmaceuticals, and nutraceuticals. However, the low solubility, chemical instability, and low bioavailability of bioactive substances limit their application in the food industry. Using nanotechnology to prepare protein nanoparticles to encapsulate and deliver active substances is a promising approach due to the abundance, biocompatibility, and biodegradability of proteins. Common protein-based nanocarriers include nano-emulsions, nano-gels, nanoparticles, and nano complexes. In this review, we give an overview of protein-based nanoparticle fabrication methods, highlighting their pros and cons. Additionally, we discuss the applications and current issues regarding the utilization of protein-based nanoparticles in the food industry. Finally, we provide perspectives on future development directions, with a focus on classifying bioactive substances and their functional properties.

## 1. Introduction

Nanotherapeutics has emerged as a promising application platform with great potential in drug delivery [1]. In recent decades, numerous nano-delivery systems have been created and refined to increase the bioavailability of drugs and enhance their therapeutic benefits. Nanoscale carrier substances are highly effective in delivering active substances to specific targets. Their small size, large surface area, and strong reactivity and adsorption capabilities render them ideal for achieving high efficiency, precision, and utilization of functional materials, as well as superior absorption, controlled release, and specific targeting of nutrients in vivo [2,3].

Bioactive substances that are present in nature generally have powerful physiological or pharmacological effects, such as curcumin [4], tocopherols [5], flavonoids [6], resveratrol [7], anthocyanins [8], puerarin [9], ferulic acid [10], and carvacrol [11], which are not essential for maintaining human growth and development but have significant health benefits [12]. Recent research has shown that bioactive substances have plenty of functions—such as antioxidant [13], anti-inflammatory [14], antibacterial [15], anticancer, and immunomodulatory [16,17] functions—and play an important role in health maintenance, disease prevention, and physiological function regulation. However, owing to their physicochemical properties, most bioactive substances are environmentally sensitive, unstable to light and heat, easily damaged or degraded, structurally altered, thus reducing or losing their activity, and have low bioavailability [18,19,20]. These factors have greatly limited their application. The construction of nanoparticles with biomolecules is an effective was to prevent the destruction of bioactive substances and to achieve better bioavailability in vivo [21,22,23].

Nanoparticles are unique entities with dimensions on the order of nanometers, which offer opportunities to develop novel carriers and materials. Nanoparticles can be made from all sorts of materials—both from natural macromolecules, such as proteins, polysaccharides, and lipids, as well as synthetic materials, such as carbon, metals, and organic and inorganic polymers—and have been widely used in medical, food, environmental and energy applications [24]. Nanoparticles can protect bioactive substances from environmental factors (such as light, heat, oxygen, and storage conditions) and regulate their release in the gastrointestinal tract, thus improving their bioavailability in the human body. In recent years, researchers have designed nano-delivery carriers, such as nano-emulsions, nano-complexes, nano-gels, nano-microcapsules, and other nanoparticles. Protein is a natural biomolecule with abundant sources; moreover, it has high biocompatibility and biosafety [25,26,27]. Protein can potentially form complex delivery systems with active molecules, polysaccharides, etc. and can allow for surface modification and the attachment of active substances and other biomolecules through hydrogen, ionic, and covalent bonding, and other associations [28,29]. Compared to manufactured materials such as metals, proteins are more generally available, have superior nutritional value and safety, may be loaded with diverse compounds, and have been widely employed for embedding bioactive molecules [30]. The variable structure of protein molecules can be fabricated into spherical, fibrous, or tubular structures for different applications. Before preparing protein nanoparticles, proteins can be modified physically, chemically, and enzymatically to make them more suitable materials. In addition, the charge, molecular conformation, and polarity of proteins can have an impact on properties such as its stability and the encapsulation ability of nanoparticles [31]. Nanoprotein-based carriers not only have nano-system characteristics when constructing the delivery system but are also naturally non-toxic and harmless and have additional nutritional value, giving them advantages in the embedding system that other nanocarriers do not offer [32].

In this paper, we review the classification, sources, and functions of bioactive substances, as well as the dilemmas they face in food fortification. Applications and studies of different types of protein (including plant and animal proteins) carrier systems (nano-emulsions, nano-gels, nanoparticles, and nano complexes) for the stabilization and delivery of biologically active substances are described. In addition, we discuss in detail various types of protein nanocarriers loaded with active substances, and their suitability and compatibility with food matrices. We also outline the preparation of protein nanoparticles, analyze the strengths and weaknesses of various methods, and highlight the application of protein nanoparticles in the food industry. The toxicity and safety of nanoparticles should also be evaluated in vivo to ensure that they can be implemented in food applications. We hope that this review can provide a reference and direction for protein-based active carriers to address major diseases and health problems in practice.

## 2. Origin, Classification, and Functional Properties of Bioactive Substances

Bioactive substances are secondary metabolites derived from various metabolic processes in living organisms, including polyphenols, vitamins, natural pigments, flavonoids, etc., in addition to nutrients such as proteins, carbohydrates, amino acids, and fats. Bioactive compounds are produced from natural products and are frequently found in fruits and vegetables, marine organisms, nuts, plant leaves, and skins. They offer a variety of beneficial physiological and pharmacological effects for humans and other animals. They have a high research and usage value and are now the hotspot for functional product development. (Table 1: Classification, sources, and functional properties of bioactive substances).

As a safe and natural food additive with many sources and at low cost, active substances have a positive impact on a variety of diseases, such as diabetes [65,66], cancer [67], autoimmune diseases [68], cardiovascular diseases [69], and neurodegenerative diseases [70]. Curcumin is a polyphenolic substance extracted from the rhizomes of plants, such as ginger and araceae, and is a rare diketone compound in the plant kingdom [71]. The anti-inflammatory activity of curcumin is not only comparable to that of steroidal and non-steroidal anti-inflammatory drugs but also has a wide range of preventive properties against diseases with a higher safety profile [72]. Studies have shown that curcumin plays an essential role in a range of neurological diseases, such as its neuroprotective effects through the protection of dopaminergic neurons, modulation of cellular autophagy, and resistance to mitochondrial damage; these effects demonstrate the potential efficacy of curcumin in the treatment of Parkinson’s [73]. In a study of a novel Drosophila model of Parkinson’s disease, Nguyen et al. found that curcumin could reduce the level of oxidative stress induced by Drosophila, ubiquitin carboxyl-terminal hydrolase gene knockdown improve motor ability, and alleviate the extent of neurodegenerative lesions in Drosophila [74]. Resveratrol may prevent and improve cardiovascular disease through its anti-apoptotic effects, anti-inflammation, lipid metabolism modulation, and oxidative stress mitigation [75]. The regular intake of resveratrol-rich foods may contribute to a healthier cardiovascular system [76]. As natural phenolic products are widely present in diets, flavonoids play a beneficial role in the fight against Alzheimer’s disease caused by the aggregation of the Amyloid-beta 42 and tau protein hyperphosphorylation [77,78].

Multiple factors limit the expression of the functional activity of bioactive substances, namely their environmentally sensitivity and susceptibility to light, heat, oxygen, and pH during processing and storage. Most bioactive substances have low bioavailability, poor stability, and are difficult to absorb by the intestine [79]. In addition, lipophilic and amphiphilic active substances are insoluble in the aqueous phase. These factors dramatically limit the development and application of bioactive substances in the food field and in functional products. However, the development of nanotechnology seems to offer a solution to this problem. An increasing number of studies have demonstrated that loading insoluble bioactive substances onto some specific nanoparticles can significantly improve their water solubility, stability, bioactivity, and bioefficacy [80,81]. Therefore, the construction of nano-delivery carriers is an effective way to heighten the bioavailability of active substances and broaden their application prospects in the food and medical industries [82].

## 3. Construction of Protein Nanoparticle Carriers

Food proteins have an outstanding capacity to bind to various active compounds, rendering them suitable raw materials for the creation of nanoparticles. With the fast growth of nanotechnology, several protein-based nanoparticle carriers, such as nanocomplexes, nano-emulsions, nanoparticles, and nanogels, have been developed (Table 2).

### 3.1. Nano-Complexes

Nanocomplexes are delivery vehicles created through interactions or self-assembling between macromolecules, such as proteins and polysaccharides, and small molecules, such as bioactive compounds. Small molecules can be bound in large molecules’ hydrophobic regions or cavities, thus effectively enhancing their solubility, stability, and bioavailability. The dispersion of the macromolecule in the nanocomplex in the solution determines the degree of solubility improvement in the small molecule’s substance. The macromolecule’s hydrolysis rate determines the small molecule’s digestibility rate. At the same time, the addition of small molecules will also have a particular effect on the properties of the main macromolecule, such as digestibility, solubility, and structural properties. The natural ligand binding sites of proteins can bind to bioactive substances with a different solubility. A range of the small molecule’s active substances have a strong protein affinity and can form complex systems with proteins with functional properties, which makes it possible to prepare protein-active molecule complexes. Kanakis et al. [93] studied the interaction between milk proteins and tea polyphenols and found that adding tea polyphenols changed the conformation of milk proteins and made the structure of the proteins more stable. The nanocomplexes constructed by ovalbumin and sodium alginate can effectively encapsulate curcumin and improve its water solubility and bioavailability [94]. YI et al. [88] fabricated whey protein isolate (WPI) sodium alginate (ALG) nanocomplexes for curcumin stabilization in beverages. Compared to free curcumin, the light and thermal stability and DPPH scavenging ability of curcumin were significantly improved in WPI–ALG nanocomplexes.

### 3.2. Nano-Emulsions

Emulsions with particle sizes of 20–200 nm are often defined as “nano-emulsions”, and they are also known as fine emulsions, ultrafine emulsions, submicron emulsions, etc. [95,96]. Compared with normal emulsions, nano-emulsions have a nanoscale particle size, more translucent emulsions, more detailed droplets, and a spherical shape; further, they exhibit good viscoelasticity at low droplet volume fractions and have higher kinetic stability and lower destabilization [97]. Proteins are amphiphilic and have superior functional characteristics such as emulsification and gelation, so they are frequently used as emulsifiers and stabilizers to improve emulsion stability [98,99]. Protein adsorption on the oil–water surface can create a stable physical barrier that improves the stability of emulsions through electrostatic repulsion and spatial potential resistance utility [100,101]. Nano-emulsion has a small volume and a relatively higher interface area, which is not only conducive to digestion but also beneficial for improving bioactive substances’ utilization rate [102,103]. Protein-stabilized oil-in-water emulsions have been widely used in the encapsulation of hydrophobic active substances, and they have a markedly protective effect on lutein [104,105], lycopene [106], lemon essential oil [107], and other active substances. The chemical breakdown rate of lutein decreased in the generated lutein-rich sodium caseinate nano-emulsions, and the nano-emulsions could stay stable after 30 days at 4 °C storage [108]. Zhao et al. [92] produced oil-in-water nano-emulsions loaded with lycopene using lactoferrin as the emulsifier and sesame oil as the oil phase. The results showed that the lycopene nano-emulsions manifested excellent stability. By simulating the gastrointestinal model, the bioaccessibility of lycopene in the nano-emulsion system was measured to be about 25%, which was significantly improved compared to free lycopene.

### 3.3. Nano-Particles

Nanoparticle delivery systems are the most common delivery systems, with the advantages of a small particle size and high bio-permeation rates. The advancement of medicine delivery systems as carriers for large and small therapeutic molecules has become a fast-growing field with great medical potential [82]. The advantages of nanoparticles prepared from proteins include good biodegradability, naturally abundant sources, low expenditure cost, and controllability. These advantages enable nanoparticles to have a broad range of applications in active substances’ encapsulation and delivery systems [109]. Various sources of proteins have been prepared into nanoparticles, including common plant proteins, such as zein, gluten, and soy protein, and some animal proteins, such as casein, collagen, and albumin. Plant proteins exhibit better loading and protection of actives and superior hydrophobicity and stability compared to traditional animal proteins [110]. Exploring newer sources of plant proteins, such as some of the less common oilseed proteins, may be a unique source of nanoparticles. Liu et al. [111] prepared zeatin–pectin nanoparticles for resveratrol (RES) loading, significantly improving RES’s oral bioavailability. Cell culture studies showed that RES in nanoparticles had stronger anti-inflammatory activity than free RES. Similarly, RES in zeatin nanoparticles had higher in vitro antioxidant and anti-tumor value-added activity [112]. Wang et al. [113] developed a novel nanoparticle with soybean isolate protein (SPI) and cellulose nanocrystals (CNC) for hydrophobic active substance curcumin (CUR) loading. It was shown that SPI–CNC nanoparticles have a small size (197.7 nm) and a high encapsulation rate (88.3%). During simulated gastrointestinal digestion, the SPI–CNC composite nanosystem exhibited a sustained release and a considerable improvement in CUR’s water solubility and chemical stability, making it a very promising nanoparticle delivery system.

### 3.4. Nano-Gels

Nanogel refers to polymer nanoparticles with a size of 1–100 nm and an inner three-dimensional network framework crosslinked by physical and chemical means of polymeric materials. The nanogel’s components have the function of a gelling agent and can be self-assembled [114]. Nanogels with a high drug loading capacity and biocompatible and responsive release properties that can easily cross the tumor vascular wall and act in cancer cells are ideal drug carriers and have attracted extensive attention from researchers [115]. Nanogel can carry out physical and chemical crosslinking through the network’s structure of protein or polysaccharide to load active substances [116]. After denaturation, proteins unfold their structures, forming cross-link and aggregating between molecules and creating gels with a three-dimensional network structure when the attractive and repulsive forces reach a balance. Depending on the treatment, they can be subdivided into chillogels and thermogels, where chillogels can be used to deliver thermosensitive active substances [117,118]. The retinol embedding rate in whey protein cold gel can reach 80%, which has good potential to protect sensitive molecules from oxidation [119]. Wang et al. [120] fabricated a new biocompatible and self-assembling acylated rapeseed protein isolate (ARPI) nanogel for curcumin delivery through chemical acylation and heat treatment-induced protein denaturation. The prepared ARPI nanogels had a particle size of 170 nm and a light-core-dark-shell spherical structure with altered spatial secondary and tertiary structures that allowed them to remain stable at a large range of pH values and ionic strengths. Aslzad et al. [121] prepared a highly efficient enzyme response composite nanogel carrier through the ion cross-linking of chitosan and gelatin, which was used to deliver doxorubicin (DOX) for breast cancer treatment. DOX was encapsulated in nanogels at a rate of around 56% and released from them in an enzymatically responsive manner. The results of cellular experiments showed that the DOX-loaded nanogels were non-toxic, had good cytocompatibility, and could be successfully absorbed by breast cancer cells, which could potentially target tumor cells. In a study by Ding et al. [122] a folic acid-loaded soy protein–soy polysaccharide composite nanogel was fabricated through heat treatment, which enhanced the performance of folic acid against unfavorable conditions such as acid, light, heat, and oxygen and expanded the application of folic acid to food and beverages (primarily acidic). Nanogels do not contain lipid-like substances, causing them to exhibit a reduced risk of oxidation and leading to a relatively more stable system.

## 4. Synthesis Strategies of Protein-Based Nanoparticles

Widespread research and development in nanotechnology have given rise to diversified strategies for fabricating nanoparticles. Proteins are typically environmentally sensitive, and salt precipitation, thermal treatment, antisolvent precipitation, and pH transformation have been successfully used to fabricate self-assembled nanoparticles. The main methods of preparing protein-based nanoparticles include antisolvent precipitation, pH-driven, salting out, and nano spray drying methods [123].

### 4.1. Anti-Solvent Precipitation

**Figure 1:**
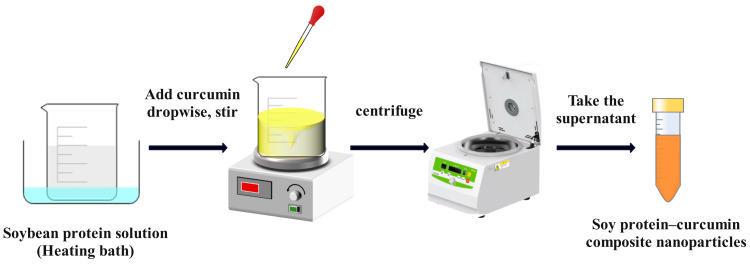
Preparation of nanoparticles using antisolvent precipitation.

An antisolvent is a substance that may dissolve in one solvent but remains insoluble in the solute in a solution system. The antisolvent precipitation method involves the addition of an antisolvent to a protein solution, which changes the polarity or charge of the protein, causing the molecules to aggregate and generate nanoparticles to precipitate out of the solution [124]. Notably, water is generally used as the antisolvent for hydrophobic proteins, whereas water is chosen as the solvent phase for hydrophilic proteins [125,126]. The construction of the delivery system through antisolvent precipitation is mainly dependent on the variation of the solvent–antisolvent ratio. The preparation process usually requires the addition of a large quantity of organic solvent, and the particle size of the fabricated nanoparticles is based on the dosage of the organic phase [127,128]. This process entails creating composite nanoparticles out of protein-based bioactive compounds. It requires dissolving high-molecular-weight proteins and low-molecular-weight active compounds in a suitable solvent, followed by vigorous mixing. Subsequently, the solvent conditions are changed to produce an antisolvent, causing co-precipitation via polarity shifts within the solution [124,126,129]. Several works have been carried out to obtain the desired physicochemical properties based on the nature of proteins through a modified antisolvent precipitation method, called thermally induced self-assembly. Thermally-induced self-assembly is a method that combines thermal treatment and antisolvent precipitation to induce the self-assembly of protein-based nanoparticles [130]. Chen et al. [131] added anhydrous ethanol dissolved with curcumin dropwise to a heat-treated (at 75–95 °C) soy protein solution, stirred and centrifuged to remove free curcumin. The supernatant obtained was soy protein–curcumin composite nanoparticles. It was shown that the solubility of curcumin in the complex was 98,000-fold higher compared to free curcumin crystals, and its stability and bioavailability were significantly enhanced. In addition, the digestibility of soy protein was improved after complexation with curcumin (Figure 1). Ebert et al. [132] prepared core–shell protein nanoparticles using an antisolvent precipitation method coupled with a continuous dual-channel micro fluidization method using a zein ethanol solution as the solvent phase and an aqueous casein solution as the antisolvent phase. By optimizing the process conditions, such as increasing the protein concentration and decreasing the ethanol content, nanoparticles with smaller particle sizes (d < 125 nm) can be obtained. The particle morphology observed via electron microscopy indicates that the core–shell protein nanoparticles are spherical. The zeatin nanoparticles fabricated via the antisolvent precipitation method, with an average particle size of 168 nm, could continuously release emamectin benzoate with superior retention and bioavailability [133].

The antisolvent precipitation method is simple and does not rely on dedicated equipment and complex operations. This approach has significant advantages, including high encapsulation efficiency, consistent size distribution, and adaptability for large-scale industrial manufacturing. As a result, it is a frequently used method for producing protein-based nanoparticles [134]. Nevertheless, this method still has some drawbacks. For example, it is difficult to select suitable solvents and counter solvents; and due to the limitations of the preparation mechanism, proteins would usually maintain the same solubility as the encapsulated compounds when embedding bioactive substances [135].

### 4.2. pH-Driven Method

The pH-driven method (pH cycling or pH transformation) is a nano-encapsulation technique that works by changing the pH of a protein solution from neutral to extreme acid/base conditions and then back to neutral or by mixing radical acid and base solutions with an ultimate pH change to neutral. During the pH change, alterations in the molecular structure and intermolecular interactions are triggered, resulting in the dissociation–repolymerization of proteins, which induces nanoparticle formation [136].

**Figure 2:**
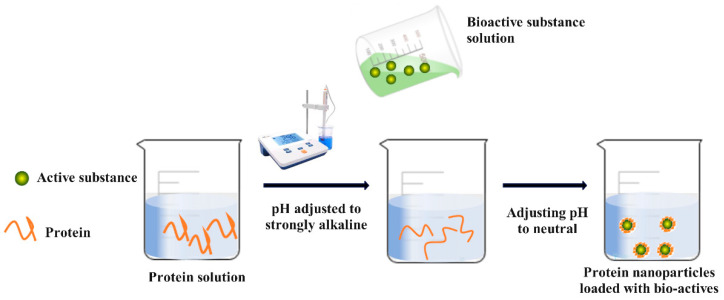
Preparation of nanoparticles via the pH-driven method.

Using the pH-driven method (Figure 2: Preparation of nanoparticles using the pH-driven method), the hydroxyl groups of soy isoflavones undergo deprotonation and a significant increase in solubility after a short period of alkaline solid treatment. When exposed to alkaline conditions, the structure of whey protein stretches and remains relatively intact. With a shift in pH to a neutral environment, soy isoflavones are converted into aggregates, and whey proteins may suffer structural rearrangement, resulting in a more compact structure between both, leading to the preparation of whey protein nanoparticles loaded with soy isoflavones [137]. The solubility of curcumin in an aqueous solution is highly pH-dependent, and the practice of protein-based curcumin nanoparticles via a pH-shift method is economically feasible and shows promising prospects [138]. Xu et al. [139] obtained acid-soluble curcumin nanoparticles by compounding soybean polysaccharides in casein using a pH transform-induced co-assembly method. The nanoparticles have excellent dispersion in acidic and neutral solutions, and the loading rate of curcumin can reach 97%. In contrast, curcumin’s physical and chemical strength is still well maintained after 30 days of storage at 25 °C. Oral administration in mice revealed a 3.4-fold increment in oral bioavailability of curcumin in nanoparticles compared to the curcumin/Tween 20 control. Pan et al. [140] dissolved sodium caseinate in deionized water, adjusted the pH to 12, and added curcumin with constant stirring. Curcumin was deprotonated and dissolved, whereas casein was dissociated. Subsequently, the pH was lowered to neutral, creating casein nanoparticles encapsulating curcumin via casein’s natural self-assembly. During preparation, curcumin was degradation-free, and the acquired curcumin composite nanoparticles displayed powerful anti-proliferative activity against colon and pancreatic cancer cells.

The pH-driven method for fabricating protein-based nanoparticles is controlled by diverse factors, such as protein concentration, protein type, the ratio of protein to active substance, sequence of solution mixing, and acidification [141]. This method is simple, green, low-energy, and organic solvent-free, which suggests that it has many applications [142].

### 4.3. Salting Out

**Figure 3:**
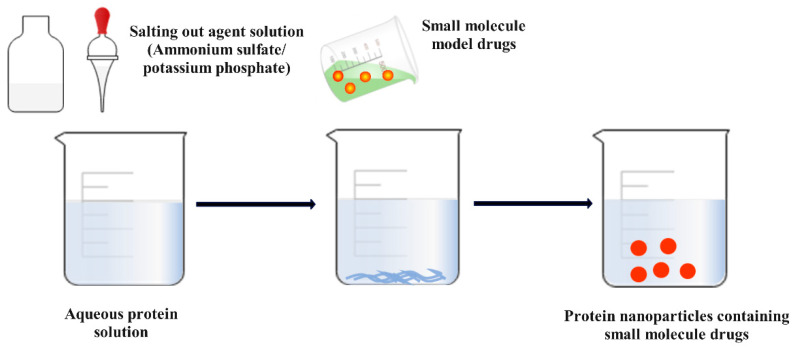
Schematic diagram of protein nanoparticles prepared via the salting out method.

Salting out is a simple approach for the fabrication of protein-based nanoparticles. In this method, by adding salting agents (such as ammonium sulfate and potassium sulfate) into a protein solution, protein molecules are induced to interact and form aggregates to obtain nanoparticles [143] (Figure 3). The principle of the salt precipitation method is based on the amphiphilic nature of proteins. The hydrophobic part of proteins can interact with water molecules to form hydrogen bonds, and after the addition of salt ions, the salt ions compete with proteins for water molecules. Moreover, with the increase in salt ion concentration, the water molecule barrier between proteins is gradually eliminated, and the hydrophobic protein–protein interaction increases, eventually leading to mutual aggregation and precipitation from the solution as nanoparticles [144]. In addition to the high drug encapsulation rate of the obtained nanoparticles, the method regulates the morphology and size of the nanoparticles. Lammel et al. [145] reported an aqueous preparation of silk fibroin nanoparticles with a controlled size and secondary structure, which were prepared by salting a silk fibroin solution with potassium phosphate. The morphology and size of the silk particles could be regulated by pH; increasing the concentration of silk protein when using 1.25 M potassium phosphate pH 8 resulted in the induction of larger particles. Based on electrostatic interactions, the silk fibroin particles can absorb and load small molecule model drugs such as crystalline violet, alcian blue, and rhodamine B and exhibit promising controlled release properties.

The salting precipitation method is appealing because of its simplicity, lack of organic solvent addition, and relatively good encapsulation efficiency. Furthermore, this approach ensures that the protein’s structure and biological function are unaffected throughout the process. Unfortunately, this method suffers from a weakness of a wide variation of the nanoparticle sizes acquired during the manufacturing process. Additionally, the purification step of nanoparticles is more tedious [146].

### 4.4. Nano Spray Drying

**Figure 4:**
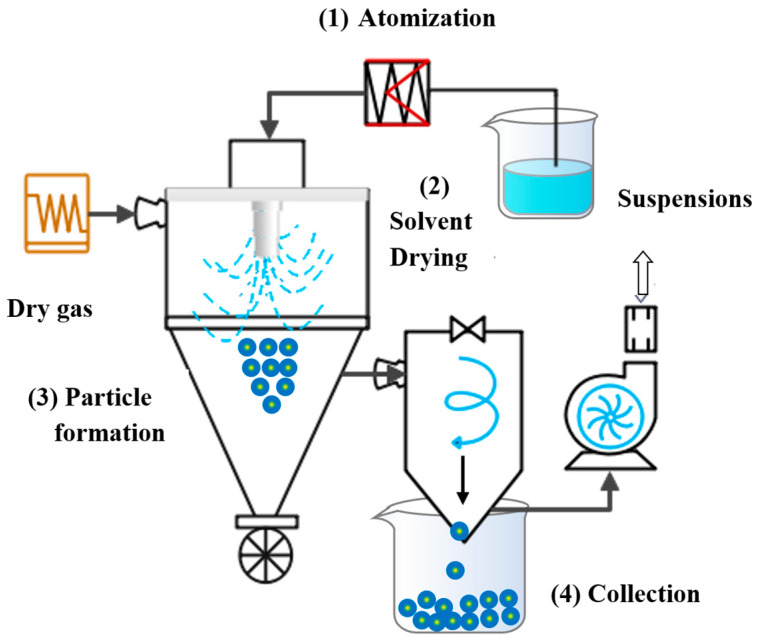
Spray drying diagram.

The nano spray drying method is an improvement in spray drying technology in which a solution containing bioactive compounds is constantly atomized into small droplets by a nozzle and then dried using hot gas to generate nanoparticles [147]. Usually, the hot gas is air, but nitrogen is also commonly used for oxygen-sensitive or flammable materials [148]. As shown in Figure 4, nano spray drying consists of the following steps: (1) atomization of the suspension with bioactive substances; (2) solvent removal and evaporation with the dried gas in the drier chamber; (3) formation of microsphere particles; and (4) separation of the particles from the gas stream and collection in a container [149,150,151]. The diameter of the particles derived from nano spray drying is usually between 0.3 and 5 μm, and the nature of the particles can be adjusted by regulating parameters such as feed flow rate, concentration of solute, temperature, atomization pressure, and nozzle diameter [152,153,154]. For example, high liquid flow rates and large nozzle diameters favor forming large particles, while small nozzle diameters and high atomization pressures lead to smaller particles. Furthermore, the increase in protein solution concentration can promote the formation of spherical nanoparticles [155]. During spray drying, the hydrophobic regions of proteins may be exposed, which causes nanoparticles to aggregate with each other. Previous studies have shown that adding surfactants (such as Tween 80) and/or increasing the protein content in a solution can suppress protein nanoparticle aggregation and promote a smoother particle surface [156,157,158]. Li et al. [159] prepared bovine serum protein nanoparticles by designing orthogonal experiments using a B-90 nano spray dryer. Scanning electron microscopy results showed the particles were smooth and spherical, with an average particle size of around 460 nm. The study confirmed that the surface of nanoparticles with the addition of Tween 80 was smoother.

In recent years, nano spray dryers capable of specifically preparing nano-microcapsules have been developed, and the resulting microcapsules have not only had good resolubility and dispersibility, but also a high encapsulation rate [160]. Compared with liquid particles, solid products produced via this method featured superior physical and chemical stability. For example, Pe’rez-Masia et al. [161] found that folic acid in whey protein microcapsules maintained almost 100% bioactive stability after 60 days of storage in a dark environment. The prime advantages of nano spray drying technology are its low cost, controllable shape and size of particles, reliable performance, and suitability for processing heat-sensitive substances. Nonetheless, to obtain a homogeneous product and achieve complete encapsulation of the active substance, a stable suspension or emulsion must be fed into the nano spray dryer.

## 5. Application of Protein-Based Nanoparticles in the Food Industry

Protein-based nanoparticles are a prospective delivery system for bioactive substances owing to their potential to encapsulate hydrophilic and hydrophobic active principles and their excellent biodegradability, non-toxicity, adhesion, and control features. Various applications of nanoparticles in food systems have been developed and investigated, such as the absorption and transport of active substances, flavors, colors, emulsion stabilizers, and functional foods. Among them, stabilizing emulsions and the production of functional foods is critical, with more concentrated research and consideration in the food industry.

### 5.1. Stabilization of Pickering Emulsions

Recently, protein-based nanoparticles have become more commonly used to stabilize emulsions. These nanoparticles have huge potential for encapsulating, transporting, and releasing hydrophobic bioactives in emulsions. Additionally, they can greatly enhance the stability of emulsions. Pickering emulsion is obtained by adsorbing solid particles at the water–oil interface as an emulsifier; however, due to the immiscibility of water and oil, the interface area increases, making this system usually unstable and hence requiring an emulsifier to be stabilized. Proteins are amphiphilic and have the ability to work as natural emulsifiers. Pickering emulsions stabilized by proteins exhibit exceptional emulsification performance, strong thermal resistance, and hydrophobicity, and therefore, manifest promising applications in the food field.

Soybean protein is known for its excellent emulsification properties. It has been found that when prepared as a stabilizer, it can significantly inhibit lipolysis [162]. Furthermore, certain active substances with antioxidant and lipid peroxidation inhibiting properties have the ability to stabilize Pickering emulsions by binding covalently to proteins. It has been shown that the composition of gliadin and proanthocyanidins can be applied as a stabilizer for Pickering emulsions and strengthens the antioxidant activity of the emulsions [163]. Ju et al. [164] prepared a Pickering emulsion stabilized by soy protein isolate-anthocyanin nanoparticles. The results revealed that the content of lipid hydrogen peroxide and malondialdehyde decreased significantly with the increase in anthocyanin; furthermore, the lower creaming index (minimum 17%) and the gradually reduced free fatty acid release rate (from 31.8% to 22.0%) suggests that the Pickering emulsion stabilized by composite nanoparticles had extraordinary oxidative stability, good emulsion stability, and extra-antibody digestibility. According to the research, protein-based nanoparticles as stabilizers for Pickering emulsions can not only inhibit the oxidation of lipids in emulsions but also replace fats in foods, which can maintain and improve the quality of foods while enhancing its healthiness. The zein–cinnamon essential oil (EO) nanoparticles not only stabilized the Pickering emulsion but also inhibited mold growth, further replacing part of the butter in the pound cake. Compared with the control group, the Pickering emulsion with a 5 g EO addition retained the color and texture of the pound cake, provided some antibacterial properties, and reduced caloric intake [165].

### 5.2. Production of Functional Foods

Numerous nutritionally active substances the human body requires, such as phenols, fatty acids, and vitamins, have powerful functional properties and present great potential in anti-inflammatory, antibacterial, anti-tumor, and hypoglycemic diseases. However, these substances are typically hydrophobic and prone to decomposition, which hinders their application in food processing and functional foods. To solve these conundrums, attempts have been made to use protein-based nanoparticles to encapsulate and protect the bioactive substances and then supplement them to food or use them as food ingredients for preparing functional foods with high nutritional value and physiological activity.

There has been growing interest in incorporating β-carotene into foods and pharmaceuticals due to its various benefits, including its ability to act as an antioxidant, scavenge free radicals, and protect cells from damage caused by lipid peroxidation. However, β-carotene has inferior water solubility and stability and is vulnerable to oxidation. Hence, encapsulating it in nanoparticles is an effective way to fulfill its application. Chuacharoen et al. [166] prepared β-carotene-encapsulated zein nanoparticles using an inverse solvent precipitation method and supplemented them to milk to obtain β-carotene-rich milk. It was shown that the zein nanoparticles improved the stability and antioxidant activity of the loaded β-carotene in milk under simulated gastrointestinal conditions and simultaneously improved the milk’s nutritional value. Curcumin is a low-molecular-weight substance with several therapeutic characteristics, but its weak water solubility and stability hinder its utilization as an effective drug. Sneharani et al. [167] studied and prepared curcumin–sunflower seed protein nanoparticles with edible ingredients to strengthen the solubility and stability of curcumin, demonstrating the feasibility of developing nutraceuticals that have enhanced its strength, solubility, and anti-inflammatory properties.

## 6. Conclusions and Prospects

The research reveals that proteins from various sources may be converted into nanoparticles using techniques such as antisolvent precipitation, pH-driven procedures, salting, and nano spray drying. Each process has benefits as well as drawbacks, and various protein types are better suited for specific preparation procedures. As a result, the best production process may be chosen depending on the qualities of the protein and the planned use. Protein-based nanoparticles contribute significantly to the utilization and protection of bioactive substances. The use of nanoparticles to encapsulate active substances and add them to foods not only solves the weaknesses of low bioavailability and easy degradation of these nutrients but also improves the nutritional value and functional properties of foods, offering a valuable idea for the development of functional foods. Additionally, certain active substances’ antibacterial and antiseptic functions allow protein nanoparticles to be used in food packaging for an extended shelf life.

Despite their distinct features, protein-based nanoparticles pose substantial obstacles in their manufacture and use. These include the insolubility of most proteins in water, which makes nanoparticle production problematic, as well as the challenges connected with establishing uniform or narrow particle sizes and assuring particle stability and preservation. However, the benefits and applications of nanoparticles extend beyond their limitations and warrant increased efforts to research and develop deeper potential. For food applications, the mechanism of protein-based nanoparticle-embedded active substances would be deeply investigated to design and construct more novel functional food products with yield-oriented goals. Moreover, predicting the release mechanism of bioactive substances and investigating the targeted delivery mechanisms of active compounds under simulated conditions in the gastrointestinal tract are the future directions of research that deserve attention and solutions.

Although several studies have demonstrated that natural protein nanoparticles are generally non-toxic and do not accumulate in the body, numerous toxicological experiments and in vivo studies should be conducted to assess their possible effects on human health and the environment in order to ensure their safety and efficacy. Lastly, protein-based nanoparticles have great promise for encapsulating and protecting bioactive compounds. Moving forward, it is critical to continue the search for optimal nanoparticle raw materials, strengthen research efforts, and investigate their application possibilities in health, food, and other important areas, in line with the use and needs of nanoparticles.

## Figures and Tables

**Table 1:** Classification, sources, and functional properties of bioactive substances.

Classification	Designation	Primary Sources	Functional Properties	Restrictions	Ref.
Polyphenols	gallic acid	Fruit, green tea, nuts, red wine	Antibacterial, anti-viral, anticancer, anti-ulcer, anti-allergy	Poor bioavailability and rapid metabolism in vivo	[33,34,35]
	chlorogenic acid	Coffee beans, apples, pears, honeysuckle	Antioxidant, anti-inflammatory, hypotensive, anti-diabetic, intestinal regulator	Easy to hydrolyze under alkaline and high-temperature environment, likely to oxidize when exposed to light and heat	[36,37]
	Curcumin	Turmeric, curry	Antibacterial, anti-inflammatory, anti-tumor, anti-liver fibrosis, hypolipidemic, anti-coagulant	Poor water solubility, low solubility, fast metabolism, easy inactivation under acid and alkaline conditions	[38,39]
	Resveratrol	Grapes, peanuts, knotweed, red wine	Antioxidant, anti-inflammatory, anticancer, heart and nervous system protection	Low water solubility, poor stability, easy to degrade under acidic or light conditions	[40,41]
Vitamins	Tocopherol	Soybean, corn, alfalfa, wheat germ oil	Antioxidant, anti-inflammatory, maintain fertility and nervous system function, improve immunity	Insoluble in water, sensitive to oxygen, unstable under alkaline conditions	[42,43]
	Retinol	Carrots, spinach, pumpkin, animal liver	Maintains visual function, regulates cell differentiation and apoptosis, and preserves epithelial tissue cell health	Poor water solubility, sensitive to light, heat, oxygen, metal ions and acidic environment, susceptible to oxidative degradation	[44,45]
	Folic acid	Green vegetables, fruits, legumes, eggs, fish	Participates in amino acid conversion and provides nutrients for cell division, a deficiency of which leads to increased risk of megaloblastic anemia, atherosclerosis and central nervous system diseases	Unstable under light (UV), heat, acidic environment, easy oxidative degradation, limited bioavailability	[46,47,48]
	Vitamin C	Fresh vegetables (potatoes, tomatoes), fruits (oranges, apples, pineapples)	Anti-oxidation, anti-scurvy, anticancer, improve human immunity, prevent and treat anemia, and participate in collagen synthesis	Unstable in aqueous solution and air, easily oxidized and degraded, vulnerable to destruction under high temperature	[49,50]
Natural pigments	β-carotene	Carrots, spinach, broccoli, soybeans, goji berries	Antioxidant, anti-inflammatory, anti-tumor, immune system, heart disease prevention	Poor water solubility, easy chemical degradation under oxygen, high temperature, and sufficient light	[51,52]
	Lutein	Corn, pumpkin, kale, orange, algae	Antioxidant, anti-inflammatory, anti-mutagenic, retinal protection, cataract retardation	Insoluble in water, sensitive to light, oxygen, and high temperature, easily degraded by oxidation	[53,54]
	Lycopene	Tomato, watermelon, grapefruit, papaya	Antioxidant, anticancer, scavenging free radicals, slowing down aging, preventing cardiovascular diseases, protecting the central nervous system	Poor water solubility and stability, easily degraded by light, heat, oxygen, metal ions and other environmental factors	[55,56,57]
Flavonoids	Quercetin	Vegetables (onions, potatoes), fruits (pomegranates, hawthorn), herbs (ginkgo biloba, mulberry leaves)	Antioxidant, antibacterial, anti-inflammatory, expectorant, cough suppressant, immune system enhancement	Slightly insoluble in water, sensible to heat and alkaline environment, weak bioavailability	[58,59,60]
	Anthocyanidin	Black wolfberry, blueberry, mulberry, grape, black fungus, black rice	Free radical scavenging, anti-inflammatory, anticancer, antibacterial	Sensitive to light, heat, and oxygen	[61,62]
	Catechin	Tea, apples, grapes chocolate	Antioxidant, anti-inflammatory, antibacterial, anti-aging, prevention of cardiovascular disease and diabetes	Unstable in aqueous solutions and neutral and acidic environments, highly susceptible to oxidation	[63,64]

**Table 2:** Construction and advantages of protein-based bioactive substance nanoparticles.

Sources	Protein Material	Nanoparticles	Particle Size	Advantages	Refs.
Plant proteins	Black bean protein	Black bean protein-quercetin nanoemulsion	278.7 nm	Smaller particle size, lower viscosity, and better emulsification performance in the compound emulsion effectively control the release of quercetin and perilla oil during gastrointestinal digestion.	[83]
	Soy protein isolate	Soybean protein isolate-1-octacosanol nanocomplex	70–100 nm	Nanocomplexes are uniformly dispersed in the aqueous phase and have excellent thermal and salt ion stability.	[84]
	Zein	Zein-sodium caseinate-xanthan gum nanocomplexes loaded with piperine	145.9 nm	Improved piperine’s water solubility and stability, significantly enhanced antioxidant activity.	[85]
	Gliadin	Curcumin-loaded gliadin-lecithin composite nanoparticles	250–280 nm	Protection of curcumin in nanoparticles from UV and heat treatment damage	[86]
	Pea protein isolate	Pea protein isolate -resveratrol nanoparticles	191.2 nm	Improves the physicochemical stability and antioxidant capacity of resveratrol	[87]
Animal proteins	Whey protein isolate	Whey-isolated protein-sodium alginate nanocomplexes loaded with curcumin	209.9 nm	The highest loading amount of curcumin in nanocomplex was 15.26 μg/mg; nanocomplexes exhibit superior stability under high sugar, salt, and high-temperature heat treatments	[88]
	Gelatin	Gelatin-procyanidin nanogel	22–138 nm	The antioxidant activity of procyanidin (PC) is protected. PC remains stable in vitro in simulated gastrointestinal digestion.	[89]
	Whey protein	Whey protein-based-fucoxanthin nanocomplex	350 nm	High fucoxanthin (FX) encapsulation rate (96.19%), enhanced FX stability to ultraviolet B, heat, NaCl, and pH, efficient FX delivery to glial cells PC12	[90]
	Casein	Casein-folic acid nanoparticles	150 nm	Protects the release of folic acid in the intestine, bioavailability of folic acid in nanoparticles is close to 52%, 50% higher than conventional aqueous solutions	[91]
	Lactoferrin	Lactoferrin-lycopene nano-emulsion	200–300 nm	Better stability, slower degradation, superior retention of lycopene, and remarkably improved bioaccessibility	[92]

## Data Availability

The data presented in this study are available on request from the corresponding author.

## References

[1] Reddy N., Rapisarda M. (2021). Properties and Applications of Nanoparticles from Plant Proteins. Materials.

[2] Dong H., Gao Y., Sinko P.J., Wu Z., Xu J., Jia L. (2016). The nanotechnology race between China and the United States. Nano Today.

[3] Yetisgin A.A., Cetinel S., Zuvin M., Kosar A., Kutlu O. (2020). Therapeutic Nanoparticles and Their Targeted Delivery Applications. Molecules.

[4] Sandhiutami N.M.D., Dewi R.S., Khairani S., Putri R.N.A. (2022). Enhancement of curcumin level and hepatoprotective effect in rats through antioxidant activity following modification into nanosized particles. Vet. World.

[5] Das Gupta S., Suh N. (2016). Tocopherols in cancer: An update. Mol. Nutr. Food Res..

[6] Lu W., Shi Y., Wang R., Su D., Tang M., Liu Y., Li Z. (2021). Antioxidant Activity and Healthy Benefits of Natural Pigments in Fruits: A Review. Int. J. Mol. Sci..

[7] Bailey H.H., Johnson J.J., Lozar T., Scarlett C.O., Wollmer B.W., Kim K., Havinghurst T., Ahmad N. (2021). A randomized, double-blind, dose-ranging, pilot trial of piperine with resveratrol on the effects on serum levels of resveratrol. Eur. J. Cancer Prev..

[8] Ayvaz H., Cabaroglu T., Akyildiz A., Pala C.U., Temizkan R., Agcam E., Ayvaz Z., Durazzo A., Lucarini M., Direito R. (2023). Anthocyanins: Metabolic Digestion, Bioavailability, Therapeutic Effects, Current Pharmaceutical/Industrial Use, and Innovation Potential. Antioxidants.

[9] Zhang W., Xu M., Wen S., Wang L., Zhang K., Zhang C., Zou H., Gu J., Liu X., Bian J. (2022). Puerarin alleviates cadmium-induced rat neurocyte injury by alleviating Nrf2-mediated oxidative stress and inhibiting mitochondrial unfolded protein response. Ecotoxicol. Environ. Saf..

[10] Drawbridge P.C., Apea-Bah F., Beta T. (2023). Bioaccessibility of ferulic acid in hulless barley varieties at stages of simulated in vitro digestion. Cereal Chem..

[11] Tavares A.G., Andrade J., Assis Silva R.R., Marques C.S., Ramos da Silva J.O., Dantas Vanetti M.C., de Melo N.R., Ferreira Soares N.d.F. (2021). Carvacrol-loaded liposome suspension: Optimization, characterization and incorporation into poly(vinyl alcohol) films. Food Funct..

[12] Yao M., McClements D.J., Xiao H. (2015). Improving oral bioavailability of nutraceuticals by engineered nanoparticle-based delivery systems. Curr. Opin. Food Sci..

[13] Lin J., Zhou W. (2018). Role of quercetin in the physicochemical properties, antioxidant and antiglycation activities of bread. J. Funct. Foods.

[14] Shamsudin N.F., Ahmed Q.U., Mahmood S., Shah S.A.A., Sarian M.N., Khattak M.M.A.K., Khatib A., Sabere A.S.M., Yusoff Y.M., Latip J. (2022). Flavonoids as Antidiabetic and Anti-Inflammatory Agents: A Review on Structural Activity Relationship-Based Studies and Meta-Analysis. Int. J. Mol. Sci..

[15] Ensafi F., Fazlyab M., Chiniforush N., Akhavan H. (2022). Comparative effects of SWEEPS technique and antimicrobial photodynamic therapy by using curcumin and nano-curcumin on Enterococcus faecalis biofilm in root canal treatment. Photodiagnosis Photodyn. Ther..

[16] Zare-Zardini H., Soltaninejad H., Ghorani-Azam A., Nafisi-Moghadam R., Haddadzadegan N., Ansari M., Saeed-Banadaki S.H., Sobhan M.R., Mozafari S., Zahedi M. (2022). Slow release curcumin-containing soy protein nanoparticles as anticancer agents for osteosarcoma: Synthesis and characterization. Prog. Biomater..

[17] Yu Z., Hong Y., Xie K., Fan Q. (2022). Research Progresses on the Physiological and Pharmacological Benefits of Microalgae-Derived Biomolecules. Foods.

[18] Tabibiazar M., Mohammadifar M.A., Roufegarinejad L., Ghorbani M., Hashemi M., Hamishehkar H. (2019). Improvement in dispersibility, stability and antioxidant activity of resveratrol using a colloidal nanodispersion of BSA-resveratrol. Food Biosci..

[19] Ubeyitogullari A., Ciftci O.N. (2019). A novel and green nanoparticle formation approach to forming low-crystallinity curcumin nanoparticles to improve curcumin’s bioaccessibility. Sci. Rep..

[20] Dini I., Grumetto L. (2022). Recent Advances in Natural Polyphenol Research. Molecules.

[21] Milincic D.D., Popovic D.A., Levic S.M., Kostic A.Z., Tesic Z.L., Nedovic V.A., Pesic M.B. (2019). Application of Polyphenol-Loaded Nanoparticles in Food Industry. Nanomaterials.

[22] Serpa Guerra A.M., Gomez Hoyos C., Andres Velasquez-Cock J., Velez Acosta L., Ganan Rojo P., Velasquez Giraldo A.M., Zuluaga Gallego R. (2020). The nanotech potential of turmeric (*Curcuma longa* L.) in food technology: A review. Crit. Rev. Food Sci. Nutr..

[23] Rai M., Ingle A.P., Pandit R., Paralikar P., Anasane N., Santos C.A.D. (2020). Curcumin and curcumin-loaded nanoparticles: Antipathogenic and antiparasitic activities. Expert Rev. Anti-Infect. Ther..

[24] Alsaba M.T., Al Dushaishi M.F., Abbas A.K. (2020). A comprehensive review of nanoparticles applications in the oil and gas industry. J. Pet. Explor. Prod. Technol..

[25] Crivelli B., Perteghella S., Bari E., Sorrenti M., Tripodo G., Chlapanidas T., Torre M.L. (2018). Silk nanoparticles: From inert supports to bioactive natural carriers for drug delivery. Soft Matter.

[26] Verma D., Gulati N., Kaul S., Mukherjee S., Nagaich U. (2018). Protein Based Nanostructures for Drug Delivery. J. Pharm..

[27] Lee E.J., Lee N.K., Kim I.-S. (2016). Bioengineered protein-based nanocage for drug delivery. Adv. Drug Del. Rev..

[28] Wang J., Li Y., Nie G. (2021). Multifunctional biomolecule nanostructures for cancer therapy. Nat. Rev. Mater..

[29] Jin S., Li S., Wang C., Liu J., Yang X., Wang P.C., Zhang X., Liang X.-J. (2014). Biosafe Nanoscale Pharmaceutical Adjuvant Materials. J. Biomed. Nanotechnol..

[30] Elzoghby A.O., Elgohary M.M., Kamel N.M. (2015). Implications of Protein- and Peptide-Based Nanoparticles as Potential Vehicles for Anticancer Drugs. Adv. Protein Chem. Struct. Biol..

[31] Zhang R., Han Y., Xie W., Liu F., Chen S. (2022). Advances in Protein-Based Nanocarriers of Bioactive Compounds: From Microscopic Molecular Principles to Macroscopical Structural and Functional Attributes. J. Agric. Food Chem..

[32] Nasery M.M., Abadi B., Poormoghadam D., Zarrabi A., Keyhanvar P., Khanbabaei H., Ashrafizadeh M., Mohammadinejad R., Tavakol S., Sethi G. (2020). Curcumin Delivery Mediated by Bio-Based Nanoparticles: A Review. Molecules.

[33] Hanieh H., Ibrahim H.-I.M., Mohammed M., Alwassil O.I., Abukhalil M.H., Farhan M. (2022). Activation of aryl hydrocarbon receptor signaling by gallic acid suppresses progression of human breast cancer in vitro and in vivo. Phytomedicine.

[34] Senapathy J.G., Jayanthi S., Viswanathan P., Umadeyi P., Nalini N. (2011). Effect of gallic acid on xenobiotic metabolizing enzymes in 1,2-dimethyl hydrazine induced colon carcinogenesis in Wistar rats—A chemopreventive approach. Food Chem. Toxicol..

[35] Soares Alves A.d.C., Mainardes R.M., Khalil N.M. (2016). Nanoencapsulation of gallic acid and evaluation of its cytotoxicity and antioxidant activity. Mater. Sci. Eng. C Mater. Biol. Appl..

[36] Jiang H., Li J., Chen L., Wang Z. (2020). Adsorption and desorption of chlorogenic acid by macroporous adsorbent resins during extraction of Eucommia ulmoides leaves. Ind. Crops Prod..

[37] Jiao W., Li X., Wang X., Cao J., Jiang W. (2018). Chlorogenic acid induces resistance against Penicillium expansum in peach fruit by activating the salicylic acid signaling pathway. Food Chem..

[38] Dizaj S.M., Sharifi S., Tavakoli F., Hussain Y., Forouhandeh H., Khatibi S.M.H., Memar M.Y., Yekani M., Khan H., Goh K.W. (2022). Curcumin-Loaded Silica Nanoparticles: Applications in Infectious Disease and Food Industry. Nanomaterials.

[39] Gayathri K., Bhaskaran M., Selvam C., Thilagavathi R. (2023). Nano formulation approaches for curcumin delivery—A review. J. Drug Deliv. Sci. Technol..

[40] Kohandel Z., Farkhondeh T., Aschner M., Pourbagher-Shahri A.M., Samarghandian S. (2021). STAT3 pathway as a molecular target for resveratrol in breast cancer treatment. Cancer Cell Int..

[41] Wang P., Luo Z.G., Xiao Z.G. (2021). Preparation, physicochemical characterization and in vitro release behavior of resveratrol-loaded oxidized gellan gum/resistant starch hydrogel beads. Carbohydr. Polym..

[42] Miyazawa T., Burdeos G.C., Itaya M., Nakagawa K., Miyazawa T. (2019). Vitamin E: Regulatory Redox Interactions. Iubmb Life.

[43] Amevor F.K., Cui Z., Ning Z., Shu G., Du X., Jin N., Deng X., Xu D., Tian Y., Zhang Y. (2022). Dietary quercetin and vitamin E supplementation modulates the reproductive performance and antioxidant capacity of aged male breeder chickens. Poult. Sci..

[44] Lee D.-U., Park H.-W., Lee S.-C. (2021). Comparing the stability of retinol in liposomes with cholesterol, beta-sitosterol, and stigmasterol. Food Sci. Biotechnol..

[45] Bento C., Matos A.C., Cordeiro A., Ramalho A. (2018). Vitamin A deficiency is associated with body mass index and body adiposity in women with recommended intake of vitamin A. Nutr. Hosp..

[46] Wusigale, Fu X., Yin X., Ji C., Cheng H., Liang L. (2021). Effects of Folic Acid and Caffeic Acid on Indirect Photo-oxidation of Proteins and Their Costabilization under Irradiation. J. Agric. Food Chem..

[47] Yang Y., Li J., Gu L., Chang C., Su Y., Liu Y., Yang Y., Dong S. (2021). Degradation of 5-methyltetrahydrofolate in model and egg yolk systems and strategies for its stabilization. J. Food Sci. Technol..

[48] Morscher R.J., Ducker G.S., Li S.H.-J., Mayer J.A., Gitai Z., Sperl W., Rabinowitz J.D. (2018). Mitochondrial translation requires folate-dependent tRNA methylation. Nature.

[49] Van der Velden U. (2020). Vitamin C and Its Role in Periodontal Diseases—The Past and the Present: A Narrative Review. Oral Health Prev. Dent..

[50] Bedhiafi T., Idoudi S., Fernandes Q., Al-Zaidan L., Uddin S., Dermime S., Billa N., Merhi M. (2023). Nano-vitamin C: A promising candidate for therapeutic applications. Biomed. Pharmacother..

[51] Kang H., Kim H. (2017). Astaxanthin and beta-carotene in Helicobacter pylori-induced Gastric Inflammation: A Mini-review on Action Mechanisms. J. Cancer Prev..

[52] Mussagy C.U., Winterburn J., Santos-Ebinuma V.C., Pereira J.F.B. (2019). Production and extraction of carotenoids produced by microorganisms. Appl. Microbiol. Biotechnol..

[53] Xu Y., Li X., Dai Z., Zhang Z., Feng L., Nie M., Liu C., Li D., Zhang M. (2023). Study on the relationship between lutein bioaccessibility and in vitro lipid digestion of nanostructured lipid carriers with different interface structures. Food Hydrocoll..

[54] Fu Y., Yang J., Jiang L., Ren L., Zhou J. (2019). Encapsulation of Lutein into Starch Nanoparticles to Improve Its Dispersity in Water and Enhance Stability of Chemical Oxidation. Starch-Starke.

[55] Pelissari J.R., Souza V.B., Pigoso A.A., Tulini F.L., Thomazini M., Rodrigues C.E.C., Urbano A., Favaro-Trindade C.S. (2016). Production of solid lipid microparticles loaded with lycopene by spray chilling: Structural characteristics of particles and lycopene stability. Food Bioprod. Process..

[56] Liu X., Dilxat T., Shi Q., Qiu T., Lin J. (2022). The combination of nicotinamide mononucleotide and lycopene prevents cognitive impairment and attenuates oxidative damage in D-galactose induced aging models via Keap1-Nrf2 signaling. Gene.

[57] Li Y., Cui Z., Hu L. (2023). Recent technological strategies for enhancing the stability of lycopene in processing and production. Food Chem..

[58] Srimathi Priyanga K., Vijayalakshmi K.J.A.J.P.C.R. (2017). Investigation of antioxidant potential of quercetin and hesperidin: An in vitro approach. Asian J. Pharm. Clin. Res..

[59] Guo Y., Bruno R.S. (2015). Endogenous and exogenous mediators of quercetin bioavailability. J. Nutr. Biochem..

[60] Lesjak M., Beara I., Simin N., Pintac D., Majkic T., Bekvalac K., Orcic D., Mimica-Dukic N. (2018). Antioxidant and anti-inflammatory activities of quercetin and its derivatives. J. Funct. Foods.

[61] Noratto G., Layosa M.A., Lage N.N., Atienza L., Ivanov I., Mertens-Talcott S.U., Chew B.P. (2020). Antitumor potential of dark sweet cherry sweet (*Prunus avium*) phenolics in suppressing xenograft tumor growth of MDA-MB-453 breast cancer cells. J. Nutr. Biochem..

[62] Husain A., Chanana H., Khan S.A., Dhanalekshmi U.M., Ali M., Alghamdi A.A., Ahmad A. (2022). Chemistry and Pharmacological Actions of Delphinidin, a Dietary Purple Pigment in Anthocyanidin and Anthocyanin Forms. Front. Nutr..

[63] Ye J.-H., Augustin M.A. (2019). Nano- and micro-particles for delivery of catechins: Physical and biological performance. Crit. Rev. Food Sci. Nutr..

[64] Yuann J.-M.P., Lee S.-Y., Yang M.-J., Huang S.-T., Cheng C.-W., Liang J.-Y. (2021). A Study of Catechin Photostability Using Photolytic Processing. Processes.

[65] Wang L., Zeng Y.-Q., Gu J.-H., Song R., Cang P.-H., Xu Y.-X., Shao X.-x., Pu L.-J., Luo H.-Y., Zhou X.-F. (2022). Novel oral edaravone attenuates diastolic dysfunction of diabetic cardiomyopathy by activating the Nrf2 signaling pathway. Eur. J. Pharmacol..

[66] Toupchian O., Abdollahi S., Salehi-Abargouei A., Heshmati J., Clark C.C.T., Sheikhha M.H., Fallahzadeh H., Mozaffari-Khosravi H. (2021). The effects of resveratrol supplementation on PPAR alpha, p16, p53, p21 gene expressions, and sCD163/sTWEAK ratio in patients with type 2 diabetes mellitus: A double-blind controlled randomized trial. Phytother. Res..

[67] Venkatas J., Daniels A., Singh M. (2022). The Potential of Curcumin-Capped Nanoparticle Synthesis in Cancer Therapy: A Green Synthesis Approach. Nanomaterials.

[68] Samsamikor M., Daryani N.E., Asl P.R., Hekmatdoost A. (2016). Resveratrol Supplementation and Oxidative/Anti-Oxidative Status in Patients with Ulcerative Colitis: A Randomized, Double-Blind, Placebo-controlled Pilot Study. Arch. Med. Res..

[69] Gal R., Deres L., Horvath O., Eros K., Sandor B., Urban P., Soos S., Marton Z., Sumegi B., Toth K. (2020). Resveratrol Improves Heart Function by Moderating Inflammatory Processes in Patients with Systolic Heart Failure. Antioxidants.

[70] Hou J., Xue J., Wang Z., Li W. (2018). Ginsenoside Rg3 and Rh2 protect trimethyltin-induced neurotoxicity via prevention on neuronal apoptosis and neuroinflammation. Phytother. Res..

[71] Ahsan R., Arshad M., Khushtar M., Ahmad M.A., Muazzam M., Akhter M.S., Gupta G., Muzahid M. (2020). A Comprehensive Review on Physiological Effects of Curcumin. Drug Res..

[72] Reolon J.B., Brustolin M., Accarini T., Vicozzi G.P., Marcondes Sari M.H., Bender E.A., Haas S.E., Sperrotto Brum M.C., Gundel A., Colome L.M. (2019). Co-encapsulation of acyclovir and curcumin into microparticles improves the physicochemical characteristics and potentiates in vitro antiviral action: Influence of the polymeric composition. Eur. J. Pharm. Sci..

[73] Abbaoui A., Gamrani H. (2018). Neuronal, astroglial and locomotor injuries in subchronic copper intoxicated rats are repaired by curcumin: A possible link with Parkinson’s disease. Acta Histochem..

[74] Thi Thanh N., My Dung V., Man Anh H., Masamitsu Y., Linh Thuoc T., Thi Phuong Thao D. (2018). Curcumin Effectively Rescued Parkinson’s Disease-Like Phenotypes in a Novel Drosophila melanogaster Model with dUCH Knockdown. Oxid. Med. Cell. Longev..

[75] Seo Y., Park J., Choi W., Ju Son D., Sung Kim Y., Kim M.-K., Yoon B.-E., Pyee J., Tae Hong J., Go Y.-M. (2019). Antiatherogenic Effect of Resveratrol Attributed to Decreased Expression of ICAM-1 (Intercellular Adhesion Molecule-1) Mechanistic Link From Focal Adhesion to Monocyte Adhesion. Arterioscler. Thromb. Vasc. Biol..

[76] Gligorijevic N., Stanic-Vucinic D., Radomirovic M., Stojadinovic M., Khulal U., Nedic O., Cirkovic Velickovic T. (2021). Role of Resveratrol in Prevention and Control of Cardiovascular Disorders and Cardiovascular Complications Related to COVID-19 Disease: Mode of Action and Approaches Explored to Increase Its Bioavailability. Molecules.

[77] Alharbi K.S., Javed Shaikh M.A., Imam S.S., Alshehri S., Ghoneim M.M., Almalki W.H., Singh S.K., Kumar D., Kumar A.P., Dua K. (2023). Role of Flavonoids in Management of Various Biological Targets in Alzheimer’s Disease: Evidence from Preclinical to Clinical Studies. Curr. Med. Chem..

[78] Dutta M.S. (2023). A study from structural insight to the antiamyloidogenic and antioxidant activities of flavonoids: Scaffold for future therapeutics of Alzheimer’s disease. Med. Chem. Res..

[79] Chen L., Cao H., Huang Q., Xiao J., Teng H. (2022). Absorption, metabolism and bioavailability of flavonoids: A review. Crit. Rev. Food Sci. Nutr..

[80] He X., Deng H., Hwang H.-m. (2019). The current application of nanotechnology in food and agriculture. J. Food Drug Anal..

[81] Singh R., Dutt S., Sharma P., Sundramoorthy A.K., Dubey A., Singh A., Arya S. (2023). Future of Nanotechnology in Food Industry: Challenges in Processing, Packaging, and Food Safety. Glob. Chall..

[82] Cho Y.-H., Jones O.G. (2019). Assembled protein nanoparticles in food or nutrition applications. Adv. Food Nutr. Res..

[83] Han L., Lu K., Zhou S., Zhang S., Xie F., Qi B., Li Y. (2021). Development of an oil-in-water emulsion stabilized by a black bean protein-based nanocomplex for co-delivery of quercetin and perilla oil. LWT Food Sci. Technol..

[84] Li D., Li X., Wu G., Li P., Zhang H., Qi X., Wang L., Qian H. (2019). The characterization and stability of the soy protein isolate/1-Octacosanol nanocomplex. Food Chem..

[85] Shirmohammadli F., Nikzad M., Ghoreyshi A.A., Mohammadi M., Poureini F. (2021). Preparation and Characterization of Zein/Sodium Caseinate/Xanthan Gum Complex for Encapsulation of Piperine and its In Vitro Release Study. Food Biophys..

[86] Yang S., Dai L., Sun C., Gao Y. (2018). Characterization of curcumin loaded gliadin-lecithin composite nanoparticles fabricated by antisolvent precipitation in different blending sequences. Food Hydrocoll..

[87] Fan Y., Zeng X., Yi J., Zhang Y. (2020). Fabrication of pea protein nanoparticles with calcium-induced cross-linking for the stabilization and delivery of antioxidative resveratrol. Int. J. Biol. Macromol..

[88] Yi J., Peng G., Zheng S., Wen Z., Gan C., Fan Y. (2021). Fabrication of whey protein isolate-sodium alginate nanocomplex for curcumin solubilization and stabilization in a model fat-free beverage. Food Chem..

[89] Carmelo-Luna F.J., Mendoza-Wilson A.M., Montfort G.R.-C., Lizardi-Mendoza J., Madera-Santana T., Gutierrez D.L., Quintana-Owen P. (2021). Synthesis and experimental/computational characterization of sorghum procyanidins-gelatin nanoparticles. Bioorg. Med. Chem..

[90] Wang C., Ren J., Song H., Chen X., Qi H. (2021). Characterization of whey protein-based nanocomplex to load fucoxanthin and the mechanism of action on glial cells PC12. LWT Food Sci. Technol..

[91] Penalva R., Esparza I., Agueros M., Gonzalez-Navarro C.J., Gonzalez-Ferrero C., Irache J.M. (2015). Casein nanoparticles as carriers for the oral delivery of folic acid. Food Hydrocoll..

[92] Zhao C., Wei L., Yin B., Liu F., Li J., Liu X., Wang J., Wang Y. (2020). Encapsulation of lycopene within oil-in-water nanoemulsions using lactoferrin: Impact of carrier oils on physicochemical stability and bioaccessibility. Int. J. Biol. Macromol..

[93] Kanakis C.D., Hasni I., Bourassa P., Tarantilis P.A., Polissiou M.G., Tajmir-Riahi H.-A. (2011). Milk β-lactoglobulin complexes with tea polyphenols. Food Chem..

[94] Feng J., Xu H., Zhang L., Wang H., Liu S., Liu Y., Hou W., Li C. (2019). Development of Nanocomplexes for Curcumin Vehiculization Using Ovalbumin and Sodium Alginate as Building Blocks: Improved Stability, Bioaccessibility, and Antioxidant Activity. J. Agric. Food Chem..

[95] Thakur P., Sonawane S., Potoroko I., Sonawane S.H. (2021). Recent Advances in Ultrasound-Assisted Synthesis of Nano-Emulsions and their Industrial Applications. Curr. Pharm. Biotechnol..

[96] Wooster T.J., Golding M., Sanguansri P. (2008). Impact of Oil Type on Nanoemulsion Formation and Ostwald Ripening Stability. Langmuir.

[97] Prastuty, Kaur G., Singh A. (2022). Shelf life extension of muffins coated with cinnamon and clove oil nanoemulsions. J. Food Sci. Technol..

[98] Zhang L., Liang R., Li L. (2022). The interaction between anionic polysaccharides and legume protein and their influence mechanism on emulsion stability. Food Hydrocoll..

[99] Niknam R., Mousavi M., Kiani H. (2022). Comprehensive evaluation of emulsifying and foaming properties of Gleditsia caspica seed galactomannan as a new source of hydrocolloid: Effect of extraction method. Food Hydrocoll..

[100] Wei R., Zhao S., Zhang L., Feng L., Zhao C., An Q., Bao Y., Zhang L., Zheng J. (2021). Upper digestion fate of citrus pectin-stabilized emulsion: An interfacial behavior perspective. Carbohydr. Polym..

[101] Yang Y., Peng W., Zhang H., Wang H., He X. (2022). The oil/water interfacial behavior of microgels used for enhancing oil recovery: A comparative study on microgel powder and microgel emulsion. Colloids Surf. A.

[102] Pandey P., Gulati N., Makhija M., Purohit D., Dureja H. (2020). Nanoemulsion: A Novel Drug Delivery Approach for Enhancement of Bioavailability. Recent Pat. Nanotechnol..

[103] Sabjan K.B., Munawar S.M., Rajendiran D., Vinoji S.K., Kasinathan K. (2020). Nanoemulsion as Oral Drug Delivery—A Review. Curr. Drug Res. Rev..

[104] Lyu Z., Wang F., Liu P., Zhang K., Sun Q., Bai X., Li A., Song X. (2021). One-pot preparation of lutein block methoxy polyethylene glycol copolymer-coated lutein nanoemulsion. Colloid Polym. Sci..

[105] Zhang Y., Kong L., Tan L. (2022). Effectiveness of nanoscale delivery systems on improving the bioavailability of lutein in rodent models: A systematic review. Crit. Rev. Food Sci. Nutr..

[106] Adriany A., Jessica S., Ana O., Raimunda S., Andreanne V., Luan S., Thiago A., Wanessa C., Maria S., Ana M. (2021). Anti-inflammatory and antioxidant activity improvement of lycopene from guava on nanoemulsifying system. J. Dispers. Sci. Technol..

[107] Zou B., Shao C., Shao L., Zhao Y., Dai R., Liu Y. (2023). Preparation of lemon essential oil nanoemulsion and its effect on the microbial community of pork patties. J. Food Sci..

[108] Li J., Guo R., Hu H., Wu X., Ai L., Wu Y. (2018). Preparation optimisation and storage stability of nanoemulsion-based lutein delivery systems. J. Microencaps..

[109] Salatin S., Jelvehgari M., Maleki-Dizaj S., Adibkia K. (2015). A sight on protein-based nanoparticles as drug/gene delivery systems. Ther. Deliv..

[110] Xu H., Yang Y. (2015). Nanoparticles derived from plant proteins for controlled release and targeted delivery of therapeutics. Nanomedicine.

[111] Liu Y., Liang X., Zou Y., Peng Y., McClements D.J., Hu K. (2020). Resveratrol-loaded biopolymer core-shell nanoparticles: Bioavailability and anti-inflammatory effects. Food Funct..

[112] Shi Q., Wang X., Tang X., Zhen N., Wang Y., Luo Z., Zhang H., Liu J., Zhou D., Huang K. (2021). In vitro antioxidant and antitumor study of zein/SHA nanoparticles loaded with resveratrol. Food Sci. Nutr..

[113] Wang S., Lu Y., Ouyang X.-k., Ling J. (2020). Fabrication of soy protein isolate/cellulose nanocrystal composite nanoparticles for curcumin delivery. Int. J. Biol. Macromol..

[114] Yang L. (2022). Nano-Hydrogel for the Treatment of Depression and Epilepsy. J. Biomed. Nanotechnol..

[115] Reeves A., Vinogradov S.V., Morrissey P., Chernin M., Ahmed M.M. (2015). Curcumin-encapsulating Nanogels as an Effective Anticancer Formulation for Intracellular Uptake. Mol. Cell. Pharmacol..

[116] Mohsenabadi N., Rajaei A., Tabatabaei M., Mohsenifar A. (2018). Physical and antimicrobial properties of starch-carboxy methyl cellulose film containing rosemary essential oils encapsulated in chitosan nanogel. Int. J. Biol. Macromol..

[117] Singhal P., Vashisht H., Nisar S., Mehra S., Rattan S. (2022). Stimulus responsive soy-protein based hydrogels through grafting HEMA for biomedical applications. Ind. Crops Prod..

[118] Abaee A., Mohammadian M., Jafari S.M. (2017). Whey and soy protein-based hydrogels and nano-hydrogels as bioactive delivery systems. Trends Food Sci. Technol..

[119] Beaulieu L., Savoie L., Paquin P., Subirade M. (2002). Elaboration and characterization of whey protein beads by an emulsification/cold gelation process: Application for the protection of retinol. Biomacromolecules.

[120] Wang Z., Zhang R.X., Zhang C., Dai C., Ju X., He R. (2019). Fabrication of Stable and Self-Assembling Rapeseed Protein Nanogel for Hydrophobic Curcumin Delivery. J. Agric. Food Chem..

[121] Aslzad S., Heydari P., Abdolahinia E.D., Amiryaghoubi N., Safary A., Fathi M., Erfan-Niya H. (2023). Chitosan/gelatin hybrid nanogel containing doxorubicin as enzyme-responsive drug delivery system for breast cancer treatment. Colloid Polym. Sci..

[122] Ding X., Yao P. (2013). Soy Protein/Soy Polysaccharide Complex Nanogels: Folic Acid Loading, Protection, and Controlled Delivery. Langmuir.

[123] Li M., Yu M. (2020). Development of a nanoparticle delivery system based on zein/polysaccharide complexes. J. Food Sci..

[124] Campos L.A.d.A., Silva Neto A.F., Noronha M.C.S., de Lima M.F., Cavalcanti I.M.F., Santos-Magalhaes N.S. (2023). Zein nanoparticles for drug delivery: Preparation methods and biological applications. Int. J. Pharm..

[125] Amoabediny G., Haghiralsadat F., Naderinezhad S., Helder M.N., Kharanaghi E.A., Arough J.M., Zandieh-Doulabi B. (2018). Overview of preparation methods of polymeric and lipid-based (niosome, solid lipid, liposome) nanoparticles: A comprehensive review. Int. J. Polym. Mater. Polym. Biomater..

[126] Martinez Rivas C.J., Tarhini M., Badri W., Miladi K., Greige-Gerges H., Nazari Q.A., Galindo Rodriguez S.A., Alvarez Roman R., Fessi H., Elaissari A. (2017). Nanoprecipitation process: From encapsulation to drug delivery. Int. J. Pharm..

[127] Joye I.J., McClements D.J. (2013). Production of nanoparticles by anti-solvent precipitation for use in food systems. Trends Food Sci. Technol..

[128] Teng Z., Luo Y., Wang Q. (2012). Nanoparticles Synthesized from Soy Protein: Preparation, Characterization, and Application for Nutraceutical Encapsulation. J. Agric. Food Chem..

[129] Xu J., Chen Y., Jiang X., Gui Z., Zhang L. (2019). Development of Hydrophilic Drug Encapsulation and Controlled Release Using a Modified Nanoprecipitation Method. Processes.

[130] Wang L., Zhang Y. (2019). Heat-induced self-assembly of zein nanoparticles: Fabrication, stabilization and potential application as oral drug delivery. Food Hydrocoll..

[131] Chen F.-P., Li B.-S., Tang C.-H. (2015). Nanocomplexation between Curcumin and Soy Protein Isolate: Influence on Curcumin Stability/Bioaccessibility and in Vitro Protein Digestibility. J. Agric. Food Chem..

[132] Ebert S., Koo C.K.W., Weiss J., McClements D.J. (2017). Continuous production of core-shell protein nanoparticles by antisolvent precipitation using dual-channel microfluidization: Caseinate-coated zein nanoparticles. Food Res. Int..

[133] Cui B., Li J., Lai Z., Gao F., Zeng Z., Zhao X., Liu G., Cui H. (2021). Emamectin benzoate-loaded zein nanoparticles produced by antisolvent precipitation method. Polym. Test..

[134] Zada M.H., Rottenberg Y., Domb A.J. (2022). Peptide loaded polymeric nanoparticles by non-aqueous nanoprecipitation. J. Colloid Interface Sci..

[135] Davidov-Pardo G., Joye I.J., McClements D.J. (2015). Food-Grade Protein-Based Nanoparticles and Microparticles for Bioactive Delivery: Fabrication, Characterization, and Utilization. Adv. Protein Chem. Struct. Biol..

[136] Dai L., Zhou H., Wei Y., Gao Y., McClements D.J. (2019). Curcumin encapsulation in zein-rhamnolipid composite nanoparticles using a pH-driven method. Food Hydrocoll..

[137] Liu Q., Sun Y., Zhang J., Zhang M., Cheng J., Guo M. (2022). Physicochemical and in vitro digestion properties of soy isoflavones loaded whey protein nanoparticles using a pH-driven method. Innov. Food Sci. Emerg. Technol..

[138] Peng S., Zhou L., Cai Q., Zou L., Liu C., Liu W., McClements D.J. (2020). Utilization of biopolymers to stabilize curcumin nanoparticles prepared by the pH-shift method: Caseinate, whey protein, soy protein and gum Arabic. Food Hydrocoll..

[139] Xu G., Li L., Bao X., Yao P. (2020). Curcumin, casein and soy polysaccharide ternary complex nanoparticles for enhanced dispersibility, stability and oral bioavailability of curcumin. Food Biosci..

[140] Pan K., Luo Y., Gan Y., Baek S.J., Zhong Q. (2014). pH-driven encapsulation of curcumin in self-assembled casein nanoparticles for enhanced dispersibility and bioactivity. Soft Matter.

[141] Korkut O.C., Ozdemir G. (2023). Encapsulation of resveratrol in rhamnolipid-zein nanoparticles using a pH-driven method: Kinetic modeling on controlled release from nanoparticles. J. Dispers. Sci. Technol..

[142] Wang Y., Zhang L., Wang P., Xu X., Zhou G. (2020). pH-shifting encapsulation of curcumin in egg white protein isolate for improved dispersity, antioxidant capacity and thermal stability. Food Res. Int..

[143] Rajendra P.K.M., Nidamanuri B.S.S., Balan A.P., Venkatachalam S., Jawahar N. (2022). A review on structure, preparation and applications of silk fibroin-based nano-drug delivery systems. J. Nanopart. Res..

[144] Jain A., Singh S.K., Arya S.K., Kundu S.C., Kapoor S. (2018). Protein Nanoparticles: Promising Platforms for Drug Delivery Applications. ACS Biomater. Sci. Eng..

[145] Lammel A.S., Hu X., Park S.-H., Kaplan D.L., Scheibel T.R. (2010). Controlling silk fibroin particle features for drug delivery. Biomaterials.

[146] Farrell L.-L., Pepin J., Kucharski C., Lin X., Xu Z., Uludag H. (2007). A comparison of the effectiveness of cationic polymers poly-L-lysine (PLL) and polyethylenimine (PEI) for non-viral delivery of plasmid DNA to bone marrow stromal cells (BMSC). Eur. J. Pharm. Biopharm..

[147] Geranpour M., Assadpour E., Jafari S.M. (2020). Recent advances in the spray drying encapsulation of essential fatty acids and functional oils. Trends Food Sci. Technol..

[148] Sosnik A., Seremeta K.P. (2015). Advantages and challenges of the spray-drying technology for the production of pure drug particles and drug-loaded polymeric carriers. Adv. Colloid Interface Sci..

[149] Fu Y.-J., Shyu S.-S., Su F.-H., Yu P.-C. (2002). Development of biodegradable co-poly(d,l-lactic/glycolic acid) microspheres for the controlled release of 5-FU by the spray drying method. Colloids Surf. B. Biointerfaces.

[150] Schafroth N., Arpagaus C., Jadhav U.Y., Makne S., Douroumis D. (2012). Nano and microparticle engineering of water insoluble drugs using a novel spray-drying process. Colloids Surf. B Biointerfaces.

[151] Bohr A., Boetker J.P., Rades T., Rantanen J., Yang M. (2014). Application of spray-drying and electrospraying/electospinning for poorly water-soluble drugs: A particle engineering approach. Curr. Pharm. Des..

[152] Rodriguez-Hernandez L.M., Araguez-Fortes Y., Pinoa J.A. (2022). Microencapsulation of vegetable oils by spray drying. Afinidad.

[153] Weng Y., Li Y., Chen X., Song H., Zhao C.-X. (2023). Encapsulation of enzymes in food industry using spray drying: Recent advances and process scale-ups. Crit. Rev. Food Sci. Nutr..

[154] Ozturk A.A., Arpagaus C. (2021). Nano Spray-Dried Drugs for Oral Administration: A Review. Assay Drug Dev. Technol..

[155] Choi K.-O., Kim D., Lim J.D., Ko S., Hong G.-P., Lee S. (2019). Functional enhancement of ultrafine Angelica gigas powder by spray-drying microencapsulation. LWT Food Sci. Technol..

[156] Wang W., Wang Y.J., Wang D.Q. (2008). Dual effects of Tween 80 on protein stability. Int. J. Pharm..

[157] Salama R.O., Traini D., Chan H.-K., Sung A., Ammit A.J., Young P.M. (2009). Preparation and Evaluation of Controlled Release Microparticles for Respiratory Protein Therapy. J. Pharm. Sci..

[158] Park C.-W., Song-Yi H., Hyun-Woo N., PureunnaraeSeo, Seung-Hwan L. (2017). Effect of Spray-drying Condition and Surfactant Addition on Morphological Characteristics of Spray-dried Nanocellulose. J. For. Environ. Sci..

[159] Lee S.H., Heng D., Ng W.K., Chan H.-K., Tan R.B.H. (2011). Nano spray drying: A novel method for preparing protein nanoparticles for protein therapy. Int. J. Pharm..

[160] Assadpour E., Jafari S.M. (2019). Advances in Spray-Drying Encapsulation of Food Bioactive Ingredients: From Microcapsules to Nanocapsules. Annu. Rev. Food Sci. Technol..

[161] Perez-Masia R., Lopez-Nicolas R., Jesus Periago M., Ros G., Lagaron J.M., Lopez-Rubio A. (2015). Encapsulation of folic acid in food hydrocolloids through nanospray drying and electrospraying for nutraceutical applications. Food Chem..

[162] Benetti J.V.M., do Prado Silva J.T., Nicoletti V.R. (2019). SPI microgels applied to Pickering stabilization of O/W emulsions by ultrasound and high-pressure homogenization: Rheology and spray drying. Food Res. Int..

[163] Zhou F.-Z., Yan L., Yin S.-W., Tang C.-H., Yang X.-Q. (2018). Development of Pickering Emulsions Stabilized by Gliadin/Proanthocyanidins Hybrid Particles (GPHPs) and the Fate of Lipid Oxidation and Digestion. J. Agric. Food Chem..

[164] Ju M., Zhu G., Huang G., Shen X., Zhang Y., Jiang L., Sui X. (2020). A novel pickering emulsion produced using soy protein-anthocyanin complex nanoparticles. Food Hydrocoll..

[165] Feng X., Sun Y., Yang Y., Zhou X., Cen K., Yu C., Xu T., Tang X. (2020). Zein nanoparticle stabilized Pickering emulsion enriched with cinnamon oil and its effects on pound cakes. LWT Food Sci. Technol..

[166] Chuacharoen T., Sabliov C.M. (2016). The potential of zein nanoparticles to protect entrapped β-carotene in the presence of milk under simulated gastrointestinal (GI) conditions. LWT Food Sci. Technol..

[167] Sneharani A.H. (2019). Curcumin-sunflower protein nanoparticles-A potential antiinflammatory agent. J. Food Biochem..

